# Complex photonic response reveals three-dimensional self-organization of structural coloured bacterial colonies

**DOI:** 10.1098/rsif.2020.0196

**Published:** 2020-05-20

**Authors:** Lukas Schertel, Gea T. van de Kerkhof, Gianni Jacucci, Laura Catón, Yu Ogawa, Bodo D. Wilts, Colin J. Ingham, Silvia Vignolini, Villads E. Johansen

**Affiliations:** 1 University of Cambridge, Department of Chemistry, Lensfield Road, Cambridge CB2 1EW, UK; 2 University Grenoble Alpes, CNRS, CERMAV, Grenoble, France; 3 Adolphe Merkle Institute, University of Fribourg, Chemin des Verdiers 4, CH-1700 Fribourg, Switzerland; 4 Hoekmine BV, Room 1.091 (iLab), Kenniscentrum Technologie en Innovatie, Hogeschool Utrecht, Heidelberglaan 7, 3584 CS, Utrecht, The Netherlands

**Keywords:** bacterial colonies, structural colour, photonic crystals, living optical material

## Abstract

Vivid colours found in living organisms are often the result of scattering from hierarchical nanostructures, where the interplay between order and disorder in their packing defines visual appearance. In the case of *Flavobacterium* IR1, the complex arrangement of the cells in polycrystalline three-dimensional lattices is found to be a distinctive fingerprint of colony organization. By combining analytical analysis of the angle-resolved scattering response of *in vivo* bacterial colonies with numerical modelling, we show that we can assess the inter-cell distance and cell diameter with a resolution below 10 nm, far better than what can be achieved with conventional electron microscopy, suffering from preparation artefacts. Retrieving the role of disorder at different length scales from the salient features in the scattering response enables a precise understanding of the structural organization of the bacteria.

## 1. Introduction

Iridescent structural colours originate from the interference of light scattered from transparent materials organized at a nanoscale level. In contrast to dye/pigmentation-based coloration originating from absorption, such structural colours allow living organisms to fully control their visual appearance both in terms of colour and angular distribution. In fact, periodic arrangements of nanostructures in one, two or three dimensions can cause very bright, angle-dependent structural colours [1], as expected for photonic crystals [2–7], while matt, homogeneous colour arises from short-range correlation of scatterers [8] or resonant scattering phenomena in disordered structures [9–11].

In the case of periodic structures, diffraction has been used to assess bacterial dimensions as long as 100 years ago [12–14] and structural colour in bacteria was noted in the literature as long ago as 1941 [15]. Recently, a number of flavobacterial strains have been reported to grow in colonies that display structural colour [16–18]. However, a detailed explanation on how the interplay between order and disorder in their packing dominates the optical appearance in this system remain unclear. Such knowledge would allow insights into bacterial colony behaviour and enables their use as living optical materials.

**Figure 1.**
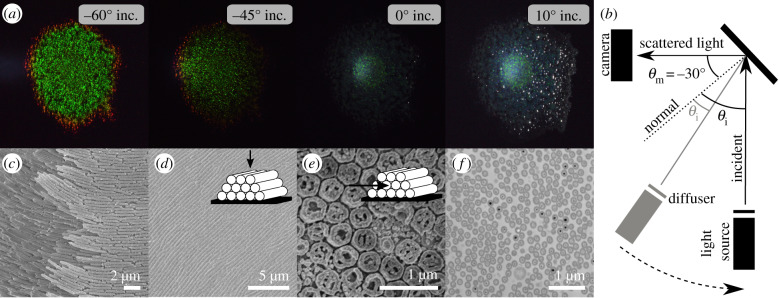
Bacterial film imaging and appearance. (*a*) Images of a bacteria colony for constant angle of observation (*θ*_m_ = −30°) but varying angle of incidence. Left to right: *θ*_i_ = −60°, −45°, 0°, 10°. (*b*) Sketch of the scattering geometry for taking images with a camera. (*c*) Scanning electron microscopy image (SEM), (*d*) cryogenic SEM top-view (inset: sketch of the geometry), (*e*) cryogenic SEM cross-section (inset: sketch of the geometry), (*f*) cross-section in transmission electron microscopy (TEM).

In this manuscript, we used the *Flavobacterium* strain IR1 (wild-type, WT) [18] as a model system to study how the 3D polycrystalline organization of rod-shaped bacteria creates structural colour (see figure 1*a*; electronic supplementary material, figure S1). Through analytical and numerical models, we assessed the lattice length of the packing with a resolution below 10 nm and estimate the value of the refractive index of their environment providing insights into the material used in their extracellular matrix. Moreover, we were able to extract information about the crystalline organization of the cells, their orientation, as well as the packing fraction by correlating all observed spectral features to physical mechanisms. Inducing deviations from the perfect crystal lattice arrangement of the bacterial positions in numerical simulations and the analytical analysis allows us to learn about the degree of disorder present on multiple length scales in the bacterial samples.

The results obtained enable further studies of the self-organizational capacity of bacterial colonies, as a thorough understanding of the structure–function relation of bacterial colonies opens new methods of modification and use of these bacteria for a range of technological applications.

## 2. Results

### 2.1. Bacterial colonies acting as photonic systems

*Flavobacterium* IR1 was cultivated on Artificial Sea Water Black (ASWB, see Methods) agar in a Petri dish. As shown in figure 1*a*, a strong angle-dependent colour appearance is observed. For small illumination angles with respect to surface normal (*θ*_*i*_ = 0°, 10°) the colony appears blue with a very low scattering intensity, while for larger angles (−45°, −60°) we can observe a strong green scattering response (see Methods for details and figure 1*b* for a schematic of the measurement geometry). In the growing edge of the colony, the colour is red-shifted, however, in the following we will consider only the centre of the colony as the growing edge continues to organize and reorganize as well as similar green appearances have been reported in earlier studies [16,18–20].

*Structural organization of bacteria.* An overview of the structural organization of IR1 bacterial colonies is obtained with a combination of electron microscopy (EM) techniques (scanning EM (SEM), cryogenic-SEM and transmission EM(TEM)) as shown in figure 1*c*–*f* . The SEM image in figure 1*c* shows an edge of the colony allowing insights into bacterial organization. The positions of the bacteria are strongly correlated in space (figure 1*d*) and this correlation can be observed across all the colony (see also electronic supplementary material in [18]). Hexagonal packing is clearly recognizable in cryo-SEM cross-section imaging (figure 1*e*, see Methods for details) and TEM (figure 1*f* , see Methods for details). However, large-area imaging both via cryo-SEM images (electronic supplementary material, figure S2a and b) and TEM (electronic supplementary material, figure S2d) reveals that the bacteria are aligned in a hexagonal lattice locally, but that there is no long-range correlation in their orientation. In fact, each domain shows local order on a length scale which is comparable to the coherence length of incidence light. Therefore, when measured on a larger scale (approx. 100 μm [18]) all possible in-(surface)plane rotations of the lattice can be expected. This observation is in agreement with the structural colour appearance that shows no angular dependency with in-plane rotation (electronic supplementary material, figure S4 in [18]) and allows us to model the bacterial colony as domains of packed bacteria with local order and random orientation on a larger scale as visualized in electronic supplementary material, figure S2c.

However, while the EM imaging techniques provide a very good qualitative description of the organization of the cells, the precise measurement of the value of the lattice constant *d* (inter bacteria distance) from these images remains challenging due to sample preparation artefacts. In fact, TEM image analysis of the structure in figure 1*f* reveals a lattice constant (inter bacteria distance) of *d* = 179 ± 17 nm, which is less than half of what we measure from the cryo-SEM cross-section (figure 1*e*,*d* = 414 ± 29 nm). Such values differ also from the cryo-SEM top view images where the lattice constant is *d* = 374 ± 36, nm if measured by a line plot (see electronic supplementary material, figure S3) and is *d* = 396 ± 36 when estimated by autocorrelation image analysis as described by Johansen *et al*. [18], where the authors obtained a value of 357 nm from a SEM top view image. Interestingly, while the absolute values of *d* differ between the different methods, the ratio between the bacterial size *a* and lattice distance *d* remains similar for all methods within errors (*a*/*d* = 0.9 ± 0.05), revealing that the artefacts are mainly connected to shrinking caused by dehydration of the colony. Cryo-SEM imaging suffers less from dehydration artefacts but quantitative values of *d* are difficult to achieve deep inside the colony, as ice crystal growth becomes an increasing issue away from the sample surface [21]. The overall sample thickness is estimated from cryo-SEM cross sections (electronic supplementary material, figure S2e) to be 10–15 μm. Moreover, as the cell orientation varies within the colony, the measured *d* is often not measured in a cross-section image that is perpendicular to the orientation of the cells. This can be seen in electronic supplementary material, figure S2d, where the same sample is imaged at different locations in cross-section leading to different absolute lattice values and sizes. On the other hand, when the structure factor of fixed colonies is evaluated from TEM and SEM images (see electronic supplementary material, figure S4), good hexagonal packing is observed. Therefore, this technique provides a very good overview of the ordering of the sample especially deep inside the colony, where the cryo-EM methods fail.

**Figure 2.**
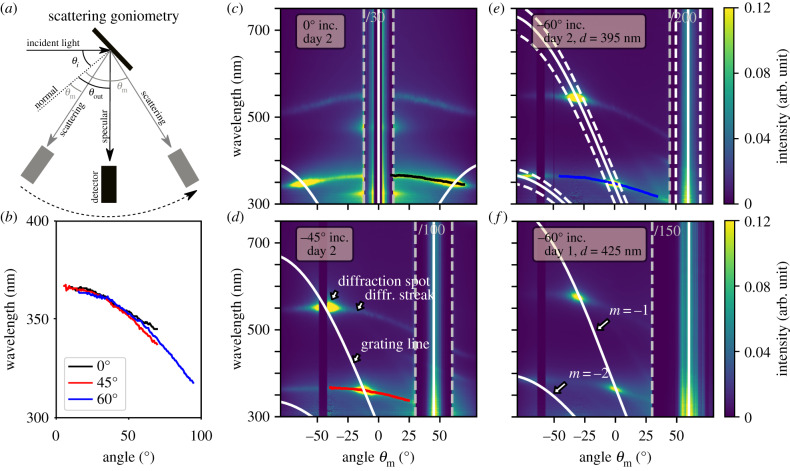
Diffraction of bacterial colonies at different scattering geometries. (*a*) Sketch of the scattering goniometry set-up. (*b*) Diffraction streak lines corrected for their incident angle extracted from (*c*) to (*e*). Scattering goniometry measurements for (*c*) *θ*_*i*_ = 0°, (*d*) −45° and (*e*) −60° incident angle for a WT IR1 sample from day 2; (*f*) −60° incident angle measurement for a WT IR1 sample from day 1. White lines indicate the analytical solution of the grating equation (equation (2.1)) of the indicated lattice constant *d*. Dashed white lines in (*e*) show the same equation indicating an uncertainty in incident angle by ± 10°.

**Figure 3.**
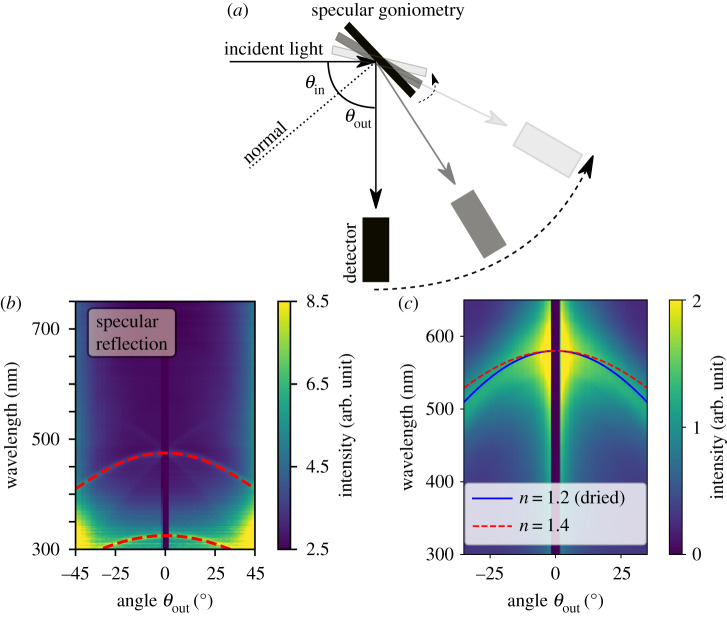
Specular reflection of bacterial colonies. (*a*) Sketch of the specular goniometry set-up. (*b*) Specular measurement for a WT IR1 sample from day 2 with an overlay of the analytical solution equation (2.2), dashed red line. (*c*) Specular measurement and fit (blue line) of a dried and fixed WT IR1 sample. Refractive index result from (*b*) shown as red dashed line for comparison.

*Goniometry as an advanced characterization tool.* In order to evaluate the value of *d* without perturbing the colony we recorded angle-resolved reflectance spectra with an optical goniometer set-up. This technique has the advantage of probing a large area of living colonies without the need for any sample preparation. Figure 2*a* shows the schematics of the geometry used in our experiment for a scattering configuration where the illumination is fixed at one angle and the scattering from the sample is measured at all the other angles (see Methods for details of the set-up). Similarly, a specular-reflection configuration can be measured (figure 3*a*), where the angle of illumination always equals the angle of observation. The specular reflection (*θ*_in_ = −*θ*_out_) is measured in the range *θ*_in_ = −45° to 45°, while the scattering properties of the sample are collected for the light incidence angles *θ*_*i*_ = 0°, −45° and −60° with respect to the sample normal. The scattering goniometry data shown in figure 2*c*–*e* together with the structural characterization by EM, will be the foundation for the subsequent analysis.

### 2.2. Analytical analysis allows structure determination

Figure 2*c*–*e* shows three scattering goniometer measurements for the light incidence angles of *θ*_*i*_ = 0°, −45° and −60° of a sample cultured for 2 days in an incubator at 25°C, measured in the centre of the colony. For the investigation, a piece of agar (approx. 2 × 2 cm) with the colony was simply cut out from the Petri dish, attached to a glass slide using double-sided tape and then mounted on the goniometer set-up. In all measurements, a region of approximately 5° around the angle of incidence shows no signal caused by the limitation in the design of the set-up (the detector arm overlaps the source). In the following logic, we aim to uncover the structural origin of all observed features by assigning them to their physical mechanisms. Small changes in the optical appearance can be detected by goniometry [18] and if related to their structural change, this will reveal changes in bacterial organization.

As expected from the EM image analysis, we observe a scattering response that can be attributed to a polycrystalline two-dimensional (2D) structure with hexagonal packing in cross-section. In our configuration, only the scattering from bacterial domains aligned perpendicular to the goniometer detector plane will contribute to the recorded scattered data (see sketch in electronic supplementary material, figure S2c). In fact, the measured angle-resolved reflection for any domain and any illumination is consistent with conical diffraction (see figs 1–3 in [22]). In more detail, in figure 2*c*–*e*, we can recognize several distinct features in the optical response of the bacterial colony. In the following, we provide a phenomenological, but quantitative description of these features derived from closed-form expressions and use this to extract the relevant parameters determining the optical response of the colony.

*Lattice constant retrieval from the diffraction spot.* Diffraction grating-like structures are formed by the bacterial colonies (see for example the cryo-SEM top view figure 1*d*). The lattice constant, corresponding to the inter-bacterial distance, can be directly obtained from the experimental goniometer data (figure 2). The angles of constructive interference from a diffraction grating can be expressed by the grating equation

2.1 $$\theta_{m}=\arcsin\left(\frac{m\lambda }{d}-\sin\theta_{i}\right),$$

where *m* ∈ [0, − 1, + 1, − 2, + 2, …] is the order, *λ* is the wavelength of light, *d* is the period of the structure, *θ*_*i*_ is the angle of incidence and *θ*_*m*_ is the reflection angle for a given order. By matching this equation with the high-intensity reflection spots in the scattering measurements (see white lines in figure 2*c*–*e*) we find that the spot location is indeed governed by the grating equation. The equation estimates the periodicity to be 395 ± 5 nm. Note that this simple method allows us to achieve very high resolution combined with high reproducibility. An uncertainty of only ± 5 nm is obtained from fitting three different WT IR1 samples on day 2 as shown in electronic supplementary material, figure S6. For comparison, a variation of Δ*d* = ± 20 nm is shown in electronic supplementary material, figure S6a as dashed white lines. This shows a remarkable degree of reproducibility for an *in vivo* measurement and confirms that goniometry can be far more accurate than TEM, SEM or cryo-SEM for obtaining averaged quantitative values of the packing.

The possibility to achieve such a high accuracy allows us to map efficiently different life stages of the bacteria colony. As an example, a lattice constant of *d* = 395 ± 5 nm was obtained for day 2 (figure 2*e*). The same sample is measured at day 1 (figure 2*f*) where the colony shows a slightly more red shifted diffraction spot, corresponding to a lattice constant of *d* = 425 ± 5 nm.

*Diffraction streak as an indication of domain tilts in the colony.* The diffraction spots in figure 2 show a certain width which can be related to a tilt of the sample surface by ± 10° (dashed white lines in figure 2*e*). This tilt in the orientation of the domains can in fact be observed in figure 1*a*, electronic supplementary material, figure S3e and S3f on the sample surface. Note that the bacteria still grow mainly as a flat film on the agar plates but with a local deviation from that is expected to occur in bacterial colonies. Similarly, earlier reports show that buckling is a common phenomenon in bacterial colonies [23–25] and calculations by Kientz *et al*. [20] suggest such effect to be present in structurally coloured bacterial colonies.

Remarkably, in the proximity of the diffraction spot, we observe a significant spread of intensity (diffraction streak). This spread stretches over the whole angular range and crosses the specular reflection even for normal incidence (*θ*_in_ = 0°) where the higher wavelength diffraction spot is out of the measured angular range. Such spread cannot be caused by tilts in the sample surface, variations in the lattice constant of the periodic arrangement of the bacteria or other types of disorder, such as orientational disorder within one crystalline unit cell. We expect this streak to originate from tilted domains within the colony. To confirm that tilted domains within the colony are responsible for the streak line of the diffraction peaks, we extracted the low wavelength scattering features in figure 2*c* (black line), *d* (red line) and *e* (blue line) and corrected them for the angle of incidence of the light. From geometrical considerations, it follows that a shift in input angle corresponds to a similar shift in output angle (electronic supplementary material, figure S5a). In figure 2*b*, we observe that the extracted scattering behaviour overlays on each other once corrected for the angle of tilt, suggesting that the streaks indeed originate from tilted domains. The observation of the spread of intensity around the diffraction spots over the whole angular range allows us to conclude that a large variety of tilts are present in the sample. Note that the diffraction spots still govern the signal intensity, meaning that most domains grow flat and parallel to the agar surface.

*Refractive index retrieval from angle-dependent specular reflection.* The optical data allows the extraction of another key parameter for photonic systems, its effective refractive index. The specular reflection peaks for a specific angle *θ*_out_ are caused by the constructive interference of waves travelling in the medium at an angle given by Snell’s Law $\theta_{2}=\arcsin(\sin\theta_{\mathrm{in}}/n_{\mathrm{avg}}),$ where *θ*_in_ is the illumination angle and *n*_avg_ is the volume average effective refractive index of the total material composite in the photonic crystal. Inside the sample, the optical path length changes with the *z*-projection of the wavevector, meaning that it is the cosine-projection of the distance the wave travels that determines the constructive interference criterion (electronic supplementary material, figure S5b). Therefore, the peak reflection wavelength *λ*_p_ for normal incidence is shifted in angular space to a shifted peak wavelength *λ*_s_ as follows:

2.2 $$\lambda_{s}=\lambda_{p}\cos(\theta_{2})=\lambda_{p}\cos\left(\arcsin\frac{\sin\theta_{\mathrm{in}}}{n_{\mathrm{avg}}}\right).$$

*λ*_p_ can be extracted from the specular measurement (figure 3*b*) by reading out the wavelength where the specular line crosses 0°. Then equation (2.2) can be used to fit *n*_avg_ to the specular reflection curve profile (red dashed lines in figure 3*b*). The fitting procedure determines the average refractive index to *n*_avg_ = 1.4 ± 0.05. This is close to the reported values of *n*_bac_ = 1.38 and *n*_agar_ = 1.34 for another *Flavobacterium* grown on an agar plate [20]. For measurements on a dried sample, we obtain *n*_avg_ ≈ 1.2 ± 0.05 (see blue line in figure 3*c*), which indeed confirms the drying out of the material compared to the live, hydrated state. We conclude that this method is a quick way to estimate or confirm the refractive index range of an unknown system, and can directly be used to investigate the composition of the extracellular matrix although more precise techniques [26,27] are needed to better resolve the refractive index precisely.

### 2.3. Numerical analysis of a crystalline bacterial arrangement

In the following, we provide a full description of the optical response from the bacterial colony by finite-element calculations. We further present a bandgap analysis of the photonic crystal structure to provide a more intuitive insight on the propagation of the electromagnetic modes inside the bacterial colony. These results uncover the physical origins of the optical response of the studied sample.

**Figure 4.**
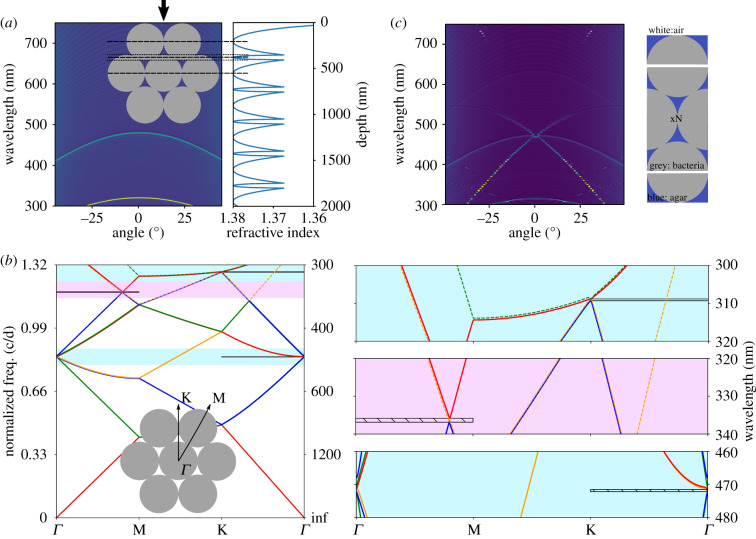
Optics of a low refractive index contrast 2D photonic crystal. (*a*) Left: multilayer 1D transfer matrix simulation with layers as indicated in the sketch (inset), black arrow indicates normal surface incidence. Right: refractive index structure used for the multilayer calculation. (*b*) MIT photonic band (MPB) simulation showing the band structure of a hexagonal crystal. Inset: sketch of the crystal with indicated direction where photonic bandgaps where found. Right: zoom ins to the photonic bandgap positions. Horizontal black (hatched) lines indicate partial band gap position. (*c*) FEM simulation of the hexagonal lattice of finite thickness with *N*=41 repeating unit cells. The repeating structure is sketched on the right.

In figure 1*c*, we observed that the bacterial stack in tightly packed layers, forming a hexagonal arrangement in cross-section (figure 1*e*,*f*). In a first approximation, the optical response can be obtained by using a 1D transfer matrix approach [28,29] (see Methods) with a refractive index structure as pictured in figure 4*a* (right). In this case, we assumed the refractive index of the bacteria to be *n*_bac_ = 1.38 and the surrounding matrix *n*_env_ = 1.34 in agreement with previous reports [20]. The angular dependency of the multilayer system is studied by changing the angle of incidence and measuring the wavelength-resolved reflectance. The result shown in figure 4*b* reveals that this simplified simulation of the 2D photonic crystal structure already predicts the specular reflection response measured experimentally.

*Bandgap analysis confirms 2D hexagonal photonic crystal.* To further understand the origin of the specular reflection and diffraction peaks, a photonic bandgap analysis on a hexagonal 2D photonic crystal structure was performed using the MPB code [30] (figure 4*b*). In this calculation, the input parameters are the lattice constant (*d* = 395 nm) and the tabulated refractive indices. The result of this analysis shows similar behaviour for both transverse electric (TE) and transverse magnetic (TM) polarization. Therefore, in this section, we will only discuss the results for the TM modes in figure 4*b* and refer to electronic supplementary material, figure S7b for the TE modes. In figure 4*b,* note that the dashed lines correspond to asymmetric modes while the solid lines correspond to symmetric modes. The symmetry of the modes was identified in electronic supplementary material, figure S7a. Asymmetric modes can be disregarded when searching for bandgaps as they cannot be excited by an incident plane wave [31].

From the bandgap diagram, three partial band gaps in the visible light range are found manually. The two bandgaps in the *Γ*K-direction are at 309 nm and 471 nm (light blue background) and correspond to the direction where light is incident perpendicular to the colony surface. The gaps are both less than 1 nm (see figure 4*b* zoom on the right), owing to the low refractive index contrast, such that mode banding cannot be excluded. Despite their small spectral width these two band gaps can still lead to a noticeable signal in the experiments as they form the origin of the specular reflection lines. It is worth noticing that the visible light reflections in the experiments originate from band gaps between higher bands and not the first band, which would require roughly twice the bacterial cell size. This seems to be different from other natural photonic crystal systems [32–34].

Another bandgap is found in the *Γ*M-direction at 336 nm (magenta background) corresponding to a 30° (or −30°) tilt of the structure. Interestingly, the appearance of one of the band gaps in the UV part of the optical spectrum may provide a functional adaptation of the bacterial colonies in providing a UV protection mechanism. To further understand the optical signal corresponding to this gap we simulated the angular resolved reflectance of a multilayer tilted by 30° and −30° approximating the hexagonal structure as sketched in electronic supplementary material, figure S8a. Although the experimental case is different, the observed features can be related to two weak signals observed in the specular measurements seen as crossing lines of the high wavelength specular line (see arrows to rescaled specular measurement in electronic supplementary material, figure S8). In the experiments, we observe that the signal coming from tilted layers with respect to the sample normal (see electronic supplementary material, figure S8a) is weakened in intensity compared to the symmetric specular lines.

*Finite-element method reveals photonic bandgap lines.* In order to confirm the parameters established from the analytical analysis and prior literature, as well as to check our predictions from the bandgap analysis and multilayer simulations, we simulated the specular and diffracted reflection using the finite-element method (FEM). Combining the FEM with a transfer matrix formalism, we can resolve Maxwell’s equations for the full system, including the air interface and the substrate interface. In this model, we only assumed a periodic, hexagonal packed bacterial colony with a period of 395 nm as extracted from equation (2.1), and the refractive indices of *n*_bac_ = 1.38, *n*_agar_ = 1.34. We use an in-house finite-element solver [35,36] combined with a transfer matrix method [37] for efficient reflection calculations of the periodic layers of bacteria, meaning that only one layer of bacteria were simulated and the solution then repeated *N* times to obtain the overall scattering characteristics (figure 4*c*). This result shows excellent agreement with the specular measurements as shown with a rescaled intensity in electronic supplementary material, figure S8c. All specular lines are recovered by the FEM modelled hexagonal packed crystal structure. Given the model assumptions, the prediction of the reflection wavelengths is fairly accurate. The reflection bandwidths are much more confined, which is to be expected for a completely periodic model system. In contrast to all earlier introduced studies, the FEM calculations allow us to study the effect of the photonic band gaps on higher order diffraction. By plotting the first-order diffraction spectra for varying angles of incidence (see electronic supplementary material, figure S8d), we find that the high wavelength diffraction giving rise to the strong green appearance of the bacterial colonies only sets in for incident angles larger than *θ*_in_ ≈ 20°.

### 2.4. Learning from disorder

In the bandgap analysis as well as the FEM study we assumed, the bacteria were forming a perfect hexagonal crystalline lattice from touching hard disks in cross-section. By contrast, in EM cross-sections (figure 1*e*,*f*), we observed that the bacteria resemble soft disks with a certain degree of packing and orientational disorder. To account for such effects we, therefore, perform finite-difference time-domain (FDTD) simulations [38,39].

**Figure 5.**
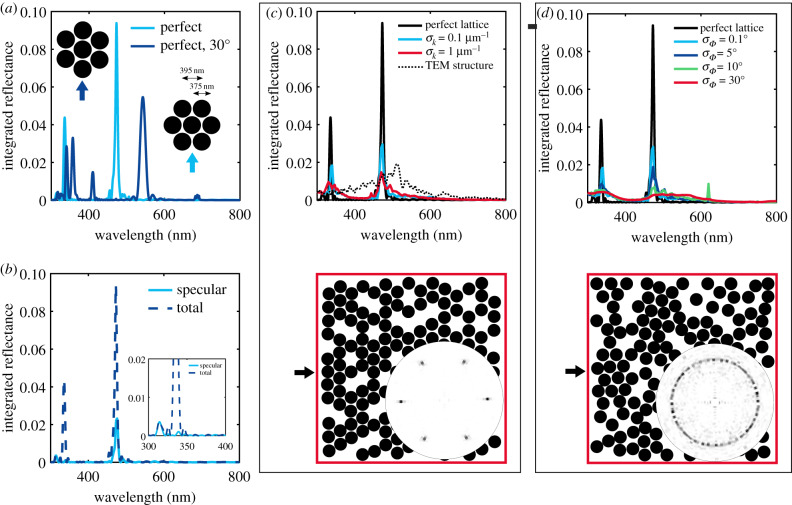
Introducing disorder in a photonic crystal lattice: FDTD simulations of spheres packed in a hexagonal lattice with diameter 375 nm, lattice distance *d* = 395 nm, filling fraction *f* = 0.6 and sample volume 5 × 5 μm (constant number of spheres). (*a*) Angular integrated reflectance for two different crystal orientations in respect to the incident light (see arrows and sketch in inset). (*b*) Specular and total integrated reflectance of the ‘perfect’ lattice from (*a*). (*c*) Integrated reflectance of the hexagonal lattice with increasing disorder in the particle distance. *σ*_*k*_ is the error (standard deviation) of the module of the wavevector *k*. Exemplary structure shown below the figure with corresponding structural correlation in angular space. For comparison, the reflectance spectrum calculated with FDTD from a structure extracted from a TEM image (see electronic supplementary material, figure S4) is shown (dashed-dotted line). (*d*) Integrated reflectance of the hexagonal lattice with increasing disorder in the particle angular orientation. *σ*_*Φ*_ is the standard deviation of the angle *Φ* relative to the incident wavevector. Exemplary structure shown below the figure with corresponding structural correlation in angular space. Incident light direction indicated by black arrows.

*Perfect lattice reveals crystalline orientation.* In all FDTD calculations, we study the integrated total reflection for normal incidence of a plane wave. As shown in figure 4, the direction of normal incidence on the hexagonal structure corresponds to the K*Γ* direction. We first investigate the effect of the orientation of the hexagonal packing in respect to the incident light. The total reflectance of the perfect hexagonal lattice with lattice constant of *d* = 395 nm is simulated for two crystal orientations in figure 5*a*.

For incidence in the K*Γ* direction, two strong peaks at 336 nm, 473 nm and one weak peak at 313 nm are obtained corresponding to experimentally observed features while the MΓ incidence leads to a spectrum with five peaks in the visible range, in contradiction to the situation observed in the experiments.

In figure 5*b*, we compare the total integrated reflectance with incidence in the K*Γ* direction with the spectrum of the specular reflection and find that the peaks at 313 nm and 473 nm correspond to the specular reflection while the peak at 336 nm correspond to the first-order diffraction in excellent agreement with the observation in the 0° incidence measurement in figure 2. Note that in the experimental spectra (figures 2 and 3), the specular reflection peaks show higher intensities than the diffraction peaks. This is not fully recovered in the numerical calculations as the boundary of the sample has not been taken into account and the intensities strongly depend on the domain thickness inside the colony assumed in the numerical calculations. Taking into account the sample boundary conditions, the domain thickness inside the bacterial colonies and the absorbing substrate in transmission is a challenging task, both to investigate experimentally and to calculate numerically, but would allow a quantitative comparison of intensities and give further insights into the bacterial assembly.

To conclude we related the features observed in the spectra to a specific orientation of the crystalline structure as pictured in figure 5*a* (right inset). The orientation found here is expected as it allows a better packing in a 2D geometry, forming a flat surface, consistent with surface energy minimization arguments.

*Disorder analysis.* To study the effect of disorder on the total integrated reflectance, we introduce lattice disorder (figure 5*c*) and crystalline orientation disorder (figure 5*d*) separately (see Methods for details). The average value of the lattice constant is assumed to be the one retrieved from the analytical analysis. We expect the rod-shaped bacteria to have a diameter smaller than this value to allow for small amounts of disorder. This claim is further strengthened by the cryo-SEM cross-section shown in figure 1*e*,*f* . In these images, we extracted averaged sizes at least 5% smaller than the distance. Therefore, in all FDTD simulations, we used a particle diameter of 375 nm while the particle distance (lattice constant) is kept at 395 nm.

In figure 5*c,* we observe that small amounts of disorder in the lattice distance (hardly visible in the simulated structure) already lead to a strong decrease in the peak intensity and a broadening of the peak width, similar to the reported literature for another 2D photonic crystal [40]. A similar effect is also observed for the disorder in angular alignment within the hexagonal crystal as introduced in figure 5*d*. Here, already small amounts of disorder can be observed in the real structure (electronic supplementary material, figure S9). Complete angular disorder (see red line and corresponding structure as well as isotropic angular space distribution in figure 5*d*) leads to a broad spectrum with no distinct peaks in the visible range. Despite the low refractive index contrast in our samples, a remarkably bright angle-dependent colour appearance is observed in the measurement. From this, we conclude that a high degree of order within each crystalline domain remains in the studied samples.

The FDTD method can further be applied to real structures imaged by TEM or cryo-SEM in cross-section. A TEM image is converted to a binary image (electronic supplementary material, Fig S4) and the total angular integrated reflectance spectrum is calculated. The result is shown in figure 5 as a black dotted line. A very broad peak around 510 nm is observed strongly deviating from the experimental results. This emphasizes that goniometry in comparison with numerical tools allows more insights into the structural arrangement of living microorganisms than EM imaging methods alone.

## 3. Conclusion

In summary, we studied the interplay between order and disorder in the structural organization of iridescent bacterial colonies. By correlating angle-resolved measurements with simple closed-form expressions, we were able to deduct the inter-cellular spacing as well as estimate the average refractive index of cells and their matrix.

The combination of analytical scattering analysis, multilayer simulations, bandgap analysis and finite-element calculations allowed us to recover the origin of all optical features observed in specular and scattering goniometry measurements. Moreover, the quantitative characterization of the broadening of the diffraction peak led us learn about the degree of disorder on domain alignment and enables further studies of bacteria communication under different growth conditions: as an example it might be useful in estimating the physical interactions of cells during adaptation of the colony structure in response to environmental conditions.

In conclusion, our *in vivo*, non-invasive method is particularly important for biofilm characterization as bacterial colonies and biofilms are known to be differentiated dynamic structures [41,42] and traditional methods rely often only on imaging studies confined to monolayer microcolonies [43] or static sectioning approaches. Additionally, our finding can be extended to any other 2D photonic tissue in other organisms as the response only depends on the size ratio between the scattering elements and the optical wavelength.

## 4. Material and methods

### 4.1. Sample isolation

Strain IR1 was isolated on ASW agar during a screening of estuarine sediment samples from the Neckarhaven region of Rotterdam harbour [18]. Strain IR1 is a yellow-pigmented Gram-negative bacterium culturable on ASW agar under aerobic conditions from 2 to 30°C with salinity matching the location where it was isolated (0.5–1.5). On ASWB plates, containing nigrosine as a contrasting agent, IR1 formed intensely structurally coloured green colonies with red/orange edges as seen in electronic supplementary material, figure S1.

### 4.2. Sample preparation

Bacteria were grown on ASWB agar plates, containing 17.5 g agar (Invitrogen), 10 g KCl, 1 g yeast extract (Sigma: Y1625), 5 g peptone (Sigma: 70 173) and 0.33 g nigrosin (Sigma: N4763) per litre. Plates were allowed to air dry for 30 min after which bacteria were added, and then air dried for another 15 min. Bacteria were inoculated on the plates by suspending them in a 1% w/v KCl solution, of which 5 μl was deposited on the plates. These plates were placed into an incubator at 25°C for 1/2 days before measuring.

### 4.3. Photographic images

A directed beam from a xenon lamp (HPX2000, Ocean Optics) was used as a light source. A white sheet was put in between the beam and the sample to simulate a diffusive, but directed illumination. All images where taken with a camera (Nikon D3200).

### 4.4. Scanning electron microscopy

The SEM studies (figure 1*c*) were performed on fixed colonies that resulted in the dried material maintaining structural colours described elsewhere (see electronic supplementary material in Johansen *et al.* [18]).

### 4.5. Cryogenic SEM

Cryo-SEM was performed on an FEI Verios 460 scanning electron microscope with a Quorum cryo-transfer system PP3010T. A piece of agar (2 × 2 cm) with bacteria was cut out and placed in a specimen holder with colloidal graphite suspension. The specimen holder plus sample was then plunge frozen in liquid ethane and transferred to a specimen preparation chamber cooled down to approximately −140°C. Samples for cross-sectional view were then freeze-fractured, all samples were sublimed at −90°C and sputter-coated with platinum. All samples were imaged at 2.00 kV acceleration voltage.

### 4.6. Transmission electron microscopy

Samples were embedded for TEM by chemical fixation. Small pieces of the bacteria colony on agar gel were cut out and fixed with glutaraldehyde (2 wt%) and formaldehyde (2 wt%) in 0.1 M sodium cacodylate buffer (pH 7.5) overnight at 4°C. During the fixation the bacteria colony had detached from the agar which was subsequently removed. Samples were then rinsed with the buffer solution and post-fixed in an OsO_4_ solution buffered at pH 7.4 for 2 h at 4°C. The samples were then washed with deionized water and dehydrated through a graded ethanol series (30–100%) to dry acetonitrile. They were left in a mixture of 50% dry acetonitrile and 50% Quetol 651 epoxy resin overnight, followed by infiltration in Quetol resin for 2 days. Samples were placed in a silicon mould with Quetol resin and cured for 2 days at 65°C. Ultrathin sections were cut using an ultramicrotome Leica Ultracut E. with a 45° diamond knife (Diatome). Sections were placed on carbon-coated copper grids and post-stained with 2 wt% uranyl acetate aqueous solution and Reynolds lead citrate solution. They were imaged in a FEI Tecnai G2 transmission electron microscope operated at 200 kV and equipped with an AMD CCD camera.

### 4.7. Goniometry

We used a xenon lamp (HPX2000, Ocean Optics) coupled through a fibre to a reflective collimator (RC08SMA-F01) as the light source in order to produce a collimated beam with a spot size of 5 mm. The same type of reflective collimator and fibre was used to couple the light to a spectrometer (AvaSpec-HS2048, Avantes) for detection. For normalization, a white diffuser was measured at 0° incidence with the detector at 5°. For further details, see [44].

### 4.8. Generation of two-dimensional structures for finite-difference time domain simulations

Structures with different degrees of disorder were generated following an inverse-design approach [39]: first, the desired number of hard particles were added to the system, then their positions were gradually changed in order to minimize the difference between the targeted structure factor and that of the ensemble of particles.

### 4.9. Finite difference time-domain simulations

The simulations were performed using LUMERICAL 8.18 (Lumerical Solutions Inc., Vancouver, BC, Canada), a commercial software based on the FDTD method.

We used a fixed number of scatterers for a simulation volume of 5 × 5 μm with a filling fraction (*ff*) of *ff* = 0.6. The value of *ff* was decided to guarantee the same number of particles both in the disordered and in the perfect hexagonal packing. Periodic boundaries conditions in the lateral direction, i.e. perpendicular to the incoming beam, *Y* and perfect matching layer (PML) boundaries in the *X* direction were used in all the calculations.

A plane incident wave is sent into the sample and the angular integrated reflection (−90° to 90°) is measured. This corresponds to an angular integration of the 0° incidence measurement in scattering goniometry (figure 2*c*). For each disorder configuration, we averaged over seven independently generated samples. In these calculations, the effect of the air–sample interface was not taken into account.

### 4.10. Finite-element method

An in-house periodic 2D finite-element solver based on [37] and implemented following the recipe in [45] was used. It has been thoroughly tested in several publications [35,36]. In the following, a short explanation of some important aspects for these particular simulations are given, but the reader is referred to [37,45] for full details.

Due to the periodic boundary conditions, the electric and magnetic field at the boundaries can be resolved by infinite sums (as opposed to integrals for the general case) of exponential basis functions multiplied by a set of coefficients. These are referred to as reflection and transmission modes at the boundaries. In the FEM implementation, this sum of modes is truncated to a reasonable number, since the lowest orders are propagation modes and higher orders are evanescent modes, whose coefficients normally tend to zero. Due to our implementation, this means that the non-periodic interfaces of the simulation model have to be of a homogeneous material in order to be described by the basis given in [37,45].

The highest computational cost in solving the FEM system is to decompose the so-called stiffness matrix in the linear FE system. It is, therefore, extremely fast to re-use this decomposition in order to solve for other combinations of transmission and reflection coefficients. By doing this, we can obtain a complete (up to the order truncation) description of transmission and reflection for any possible combination of incident waves (on both sides) [37]. This is represented in a transfer matrix, and such matrices can be combined to represent combined geometries. As a side note, one has to remember to calculate incident angles based on Snell’s law when doing this (not mentioned in [37]). Therefore, by dividing the bacteria model as seen in the sketch in figure 4*c*, we have an extremely fast way of calculating light interaction through just one period of bacteria, and then extending the results to 2*N* number of repeated bacterial cells in the depth direction. Compared to the bandgap analysis, we therefore include the effects at the interfaces (in particular the top interface, where the plane wave couples to the bacterial photonic crystal) and the effect of a finite height of the bacterial colony. Furthermore, the approach reduces computational time dramatically (compared to a full model) and makes it feasible to use a very fine resolution (Δ*λ* = 2 nm)—and also to include different incident angles. All FEM simulations for this paper were performed on a laptop computer from 2015 with two physical Intel i7 3.1 GHz cores, taking less than 24 h.

The approach has a few limitations. Firstly, the scattering matrices may become imprecise or unstable due to large differences in numerical values and since matrix inversions are needed [37]. Secondly, the interfaces have to be homogeneous (so we cannot make the bacterium smaller than the period). In practice, this means that some numeric values may be slightly off compared to full models, but we found the differences to be insignificant. The scattering matrix approach was verified by simulating the reflecting of *N* repetitions for one wavelength using a full model for low numbers of *N*, and then compared to the transfer matrix result. A deviation of less than 1% was generally observed.

### 4.11. Photonic bandgap calculations

Bandgap solving was performed using the open-source software MPB [30]. A 2D model was run for both TE and TM modes. The ctm file and post-processing scripts for reproducing the results can be found in the data repository accompanying the paper. Since a plane wave can only couple to symmetric modes [31], we needed to detect symmetric and anti-symmetric modes. This was done manually by plotting the field of the modes and search for symmetries (electronic supplementary material, figure S8).

### 4.12. One-dimensional transfer matrix method

The multilayer simulations were performed by using a 1D transfer matrix method python package [46]. It simulates light propagation in planar multilayer thin films, including the effects of multiple internal reflections and interference.

## Supplementary Material

Supplementary information: Complex photonic response reveals 3D self-organization of structural colored bacterial colonies

## Data accessibility

All data needed to evaluate the conclusions in the paper are present in the paper and/or the electronic supplementary material and/or available at the University of Cambridge data repository (https://doi.org/10.17863/CAM.46768).

## References

[1] Kinoshita S 2008 Structural colors in the realm of nature. Osaka, Japan: World Scientific Publishing Company.

[2] Yablonovitch E 1987 Inhibited spontaneous emission in solid-state physics and electronics. Phys. Rev. Lett. 58, 2059–2062. (10.1103/PhysRevLett.58.2059) 10034639

[3] Yablonovitch E, Gmitter TJ 1989 Photonic band structure: the face-centered-cubic case. Phys. Rev. Lett. 63, 1950–1953. (10.1103/PhysRevLett.63.1950) 10040722

[4] Kinoshita S, Yoshioka S, Miyazaki J 2008 Physics of structural colors. Rep. Prog. Phys. 71, 30 (10.1088/0034-4885/71/7/076401)

[5] Kinoshita S 2013 Bionanophotonics: an introductory textbook, 1st edn Singapore, Singapore: Pan Stanford Publishing.

[6] Onelli OD, van de Kamp T, Skepper JN, Powell J, dos Santos Rolo T, Baumbach T, Vignolini S 2017 Development of structural colour in leaf beetles. Sci. Rep. 7, 1373 (10.1038/s41598-017-01496-8) 28465577 PMC5430951

[7] Seago AE, Brady P, Vigneron J-P, Schultz TD 2009 Gold bugs and beyond: a review of iridescence and structural colour mechanisms in beetles (coleoptera). J. R. Soc. Interface 6(Suppl. 2), S165–S184. (10.1098/rsif.2008.0354.focus) 18957361 PMC2586663

[8] Forster JD *et al.* 2010 Biomimetic isotropic nanostructures for structural coloration. Adv. Mater. 22, 2939–2944. (10.1002/adma.200903693) 20414884

[9] Noh H, Liew SF, Saranathan V, Mochrie SGJ, Prum RO, Dufresne ER, Cao H 2010 How noniridescent colors are generated by quasi-ordered structures of bird feathers. Adv. Mater. 22, 2871–80. (10.1002/adma.200903699) 20401903

[10] Johansen VE, Onelli OD, Steiner LM, Vignolini S 2017 Photonics in nature: from order to disorder. Cham, Switzerland: Springer International Publishing.

[11] Schertel L, Siedentop L, Meijer J-M, Keim P, Aegerter CM, Aubry GJ, Maret G 2019 The structural colors of photonic glasses. Adv. Opt. Mater. 7, 1900442 (10.1002/adom.201900442)

[12] Pijper A 1943 The diffraction micrometer. S Afr. Med. J. 17, 276–282.

[13] Pijper A 1923 Diffraction in biological structures. I. The structure of colonies of the coli-typhoid group. S Afr. Med. Rec. 17, 243–248.

[14] Ponder E 1934 Diffraction patterns produced by bacteria. J. Exp. Biol. 11, 54–7.

[15] Stanier RY 1941 Studies on marine agar-digesting bacteria. J. Bacteriol. 42, 527–559. (10.1128/JB.42.4.527-559.1941) 16560467 PMC374774

[16] Kientz B, Vukusic P, Stephen L, Rosenfeld E 2012 Iridescence of a marine bacterium and classification of prokaryotic structural colors. Appl. Environ. Microbiol. 78, 2092–2099. (10.1128/AEM.07339-11) 22267664 PMC3302594

[17] Kientz B, Ducret A, Luke S, Vukusic P, Mignot T, Rosenfeld E 2012 Glitter-like iridescence within the bacteroidetes especially *Cellulophaga* spp.: optical properties and correlation with gliding motility. PLoS ONE 7, 1–12. (10.1371/journal.pone.0052900) PMC3531331 23300811

[18] Johansen VE, Catón L, Hamidjaja R, Oosterink E, Wilts BD, Rasmussen TS, Sherlock MM, Ingham CJ, Vignolini S 2018 Genetic manipulation of structural color in bacterial colonies. Proc. Natl Acad. Sci. USA 115, 2652–2657. (10.1073/pnas.1716214115) 29472451 PMC5856530

[19] Kientz B, Agogué H, Lavergne C, Marié P, Rosenfeld E 2013 Isolation and distribution of iridescent *Cellulophaga* and other iridescent marine bacteria from the Charente-Maritime coast, French Atlantic. Syst. Appl. Microbiol. 36, 244–251. (10.1016/j.syapm.2013.02.004) 23623798

[20] Kientz B, Luke S, Vukusic P, Péteri R, Beaudry C, Renault T, Simon D, Mignot T, Rosenfeld E 2016 A unique self-organization of bacterial sub-communities creates iridescence in *Cellulophaga lytica* colony biofilms. Sci. Rep. 6, 11 (10.1038/srep19906) 26819100 PMC4730217

[21] Ryan KP, Bald WB, Neumann K, Simonsberger P, Purse DH, Nicholson DN 1990 Cooling rate and ice-crystal measurement in biological specimens plunged into liquid ethane, propane, and freon 22. J. Microsc. 158, 365–378. (10.1111/j.1365-2818.1990.tb03008.x) 2395171

[22] Harvey JE, Krywonos A 2006 Radiance: The natural quantity for describing diffraction and propagation. In *The nature of light: light in nature* (ed. K Creath), Proc. of SPIE, pp. 628503–12.

[23] Boyer D, Mather W, Mondragón-Palomino O, Orozco-Fuentes S, Danino T, Hasty J, Tsimring LS 2011 Buckling instability in ordered bacterial colonies. Phys. Biol. 8, 026008 (10.1088/1478-3975/8/2/026008) 21358041 PMC3767764

[24] Smith WPJ, Davit Y, Osborne JM, Kim W, Foster KR, Pitt-Francis JM 2017 Cell morphology drives spatial patterning in microbial communities. Proc. Natl Acad. Sci. USA 114, E280–E286. (10.1073/pnas.1613007114) 28039436 PMC5255625

[25] Si T, Ma Z, Tang JX 2018 Capillary flow and mechanical buckling in a growing annular bacterial colony. Soft Matter 14, 301–311. (10.1039/C7SM01452J) 29260829

[26] Politch J, Segal M 1978 Holographic measurements of refractive-index changes. Opt. Lett. 3, 33–35. (10.1364/OL.3.000033) 19684687

[27] Stavenga DG, Leertouwer HL, Wilts BD 2013 Quantifying the refractive index dispersion of a pigmented biological tissue using Jamin-Lebedeff interference microscopy. Light: Sci. Appl. 2, e100–e100 (10.1038/lsa.2013.56)

[28] Harbecke B 1986 Coherent and incoherent reflection and transmission of multilayer structures. Appl. Phys. B: Lasers Opt. 39, 165–170. (10.1007/BF00697414)

[29] Byrnes SJ 2016 Multilayer optical calculations. arXiv:1603.02720. (http://arxiv.org/abs/1603.02720)

[30] Johnson SG, Joannopoulos JD 2001 Block-iterative frequency-domain methods for Maxwell’s equations in a planewave basis. Opt. Express 8, 173–190. (10.1364/OE.8.000173) 19417802

[31] Sakoda K 2001 Optical properties of photonic crystals. Springer series in optical sciences Berlin, Germany: Springer.

[32] Zi J, Yu X, Li Y, Hu X, Xu C, Wang X, Liu X, Fu R 2003 Coloration strategies in peacock feathers. Proc. Natl Acad. Sci. USA 100, 12576–78. (10.1073/pnas.2133313100) 14557541 PMC240659

[33] Teyssier J, Saenko SV, van der Marel D, Milinkovitch MC 2015 Photonic crystals cause active colour change in chameleons. Nat. Commun. 6, 6368 (10.1038/ncomms7368) 25757068 PMC4366488

[34] Saranathan V, Osuji CO, Mochrie SGJ, Noh H, Narayanan S, Sandy A, Dufresne ER, Prum RO 2010 Structure, function, and self-assembly of single network gyroid (*I*4_1_32) photonic crystals in butterfly wing scales. Proc. Natl Acad. Sci. USA 107, 11676–81. (10.1073/pnas.0909616107) 20547870 PMC2900708

[35] Song B, Johansen VE, Sigmund O, Shin JH 2017 Reproducing the hierarchy of disorder for *Morpho*-inspired, broad-angle color reflection. Sci. Rep. 7, 8 (10.1038/s41598-017-00061-7) 28387328 PMC5384085

[36] Andkjær J, Johansen VE, Friis KS, Sigmund O 2014 Inverse design of nanostructured surfaces for color effects. J. Opt. Soc. Am. B 31, 164–74. (10.1364/JOSAB.31.000164)

[37] Dossou K, Byrne MA, Botten LC 2006 Finite element computation of grating scattering matrices and application to photonic crystal band calculations. J. Comput. Phys. 219, 120–43. (10.1016/j.jcp.2006.03.029)

[38] Lumerical Inc. Fdtd: 3d electromagnetic simulator. See https://www.lumerical.com/products/.

[39] Jacucci G, Bertolotti J, Vignolini S 2019 Role of anisotropy and refractive index in scattering and whiteness optimization. Adv. Opt. Mater. 7, 1900980 (10.1002/adom.201900980)

[40] Stavenga DG, van der Kooi CJ, Wilts BD 2017 Structural coloured feathers of mallards act by simple multilayer photonics. J. R. Soc. Interface 14, 20170407 (10.1098/rsif.2017.0407) 28768883 PMC5582130

[41] Kim W, Levy SB, Foster KR 2016 Rapid radiation in bacteria leads to a division of labour. Nat. Commun. 7, 10508 (10.1038/ncomms10508) 26852925 PMC4748119

[42] Shapiro JA 1998 Thinking about bacterial populations as multicellular organisms. Annu. Rev. Microbiol. 52, 81–104. (10.1146/annurev.micro.52.1.81) 9891794

[43] Duvernoy M-C *et al.* 2018 Asymmetric adhesion of rod-shaped bacteria controls microcolony morphogenesis. Nat. Commun. 9, 1120 (10.1038/s41467-018-03446-y) 29549338 PMC5856753

[44] Vignolini S, Moyroud E, Glover BJ, Steiner U 2013 Analysing photonic structures in plants. J. R. Soc. Interface 10, 20130394 (10.1098/rsif.2013.0394) 23883949 PMC3758000

[45] Fuchi K, Diaz AR, Rothwell E, Ouedraogo R, Temme A 2010 Topology optimization of periodic layouts of dielectric materials. Struct. Multidiscip. Optim. 42, 483–93. (10.1007/s00158-010-0522-x)

[46] Byrnes SJ 2018 Transfer-matrix method for optics of thin and thick multilayer films. See https://github.com/sbyrnes321/tmm.

