# Supplementary material for: Complex photonic response reveals three-dimensional self-organization of structural coloured bacterial colonies
Source: J R Soc Interface. 2020 May 20;17(166):20200196. doi: 10.1098/rsif.2020.0196 (PMC7276552; doi:10.1098/rsif.2020.0196)
Supplement: Supplementary information: Complex photonic response reveals 3D self-organization of structural colored bacterial colonies [file rsif20200196supp1.pdf]

Supplementary information: Complex photonic response reveals 3D self-organization of structural colored bacterial colonies

Lukas Schertel,[1] Gea T van de Kerkhof,[1] Gianni Jacucci,[1]
Laura Catón,[1] Yu Ogawa,[2] Bodo D Wilts,[3] Colin J Ingham,[3]
Silvia Vignolini,[1*] Villads E Johansen,[1]

[1]University of Cambridge, Department of Chemistry, Lensfield Road, Cambridge, CB2 1EW, United Kingdom
[2]University Grenoble Alpes, CNRS, CERMAV Grenoble, France

[3]Adolphe Merkle Institute, University of Fribourg, Chemin des Verdiers 4, CH-1700 Fribourg, Switzerland

[4]Hoekmine BV, Room 1.091 (iLab), Kenniscentrum Technologie en Innovatie, Hogeschool Utrecht, Heidelberglaan 7, 3584 CS, Utrecht, The Netherlands

[*]Corrisponding author; E-mail: sv319@cam.ac.uk

This document contains all supplementary information associated with the manuscript named *Complex photonic response reveals 3D self-organization of structural colored bacterial colonies* by Schertel *et al.*

This PDF contains the following files:

Fig. S1: Macroscopic sample images
Fig. S2: Electron Microscopy of bacteria colonies
Fig. S3: Bacteria spacing and colony surface roughness
Fig. S4: Structure factor analysis
Fig. S5: Setup and sample sketches
Fig. S6: Lattice constant reproducibility
Fig. S7: Transverse Magnetic and Electric modes
Fig. S8: Hexagonal crystal analysis
Fig. S9: Disorder analysis

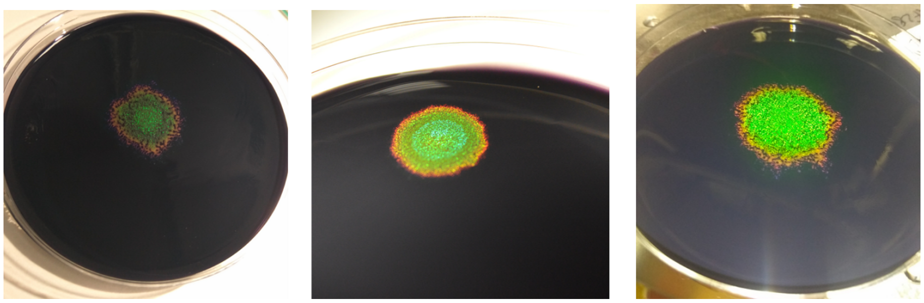

Figure S1: **Macroscopic sample images:** Three macroscopic images of a *Flavobacterium* IR1 colony at day 2 illuminated under varying light conditions in the lab.

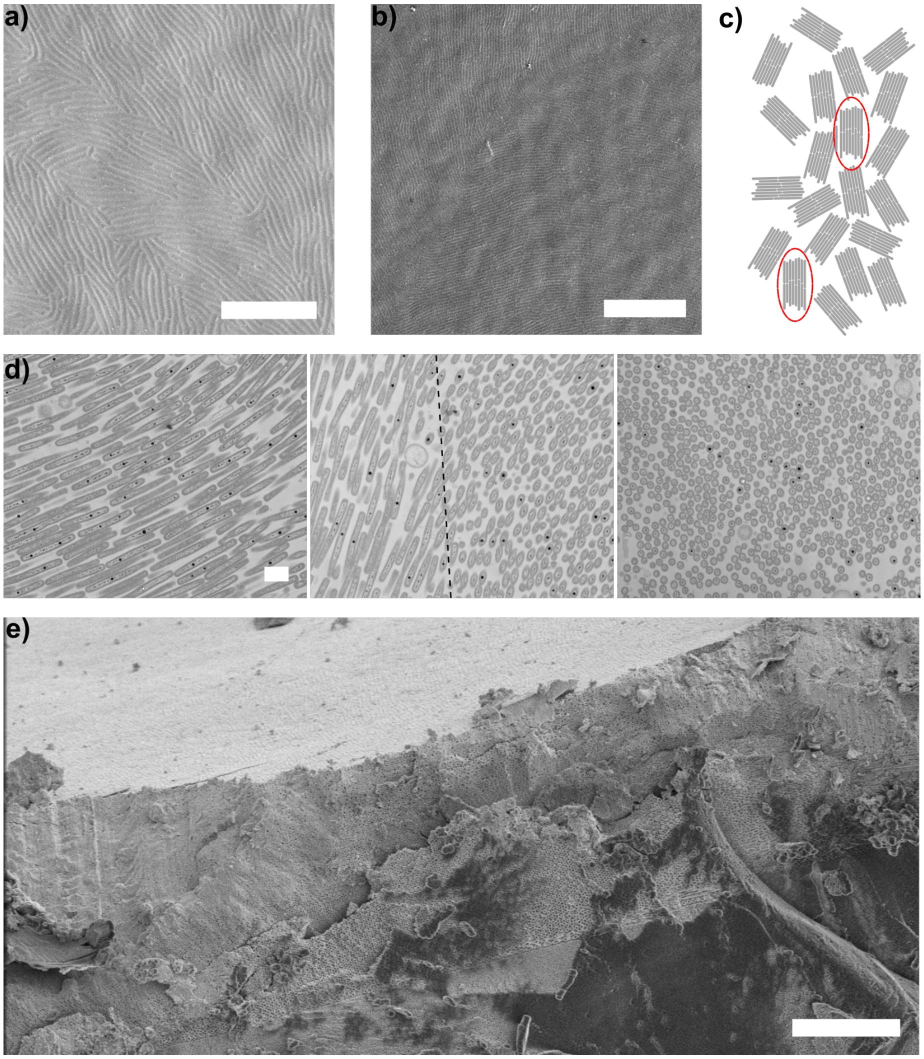

Figure S2: **Electron Microscopy of bacteria colonies:** a) Cryo-SEM of a less ordered section of a WT IR1 colony from top view. Scale bar: 5μm, b) Cryo-SEM of a very ordered section of a WT colony from top view. Scale bar: 10μm, c) Sketch of the in plane rotation of the bacterial colonies from top view. d) Three different regions of a TEM cross-section of a WT colony showing the change of orientation in-plane. Black dashed line indicates the boundary between two bacterial orientations. Scale bar: 500 nm. e) Cryo-SEM cross-section showing the overall film thickness. Scale bar: 10μm.

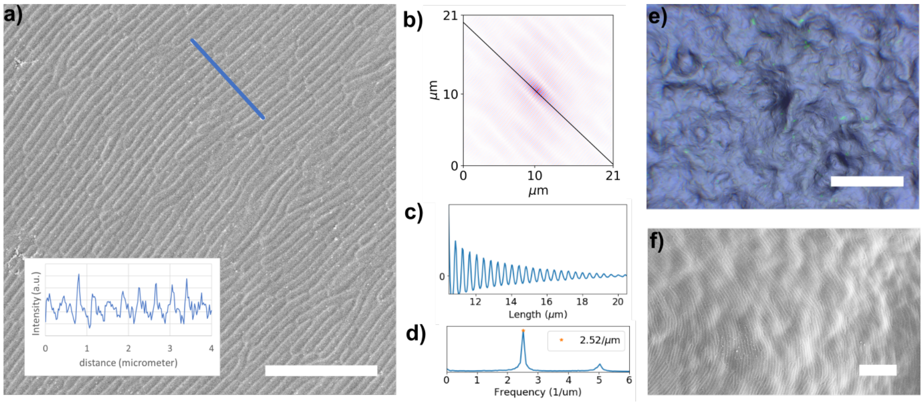

Figure S3: **Bacteria spacing and colony surface roughness:**a) Cryo-SEM of a WT colony from top view. Scale bar 5μm. Inset: Intensity profile along blue line extracted in ImageJ. b) FFT-image of the image shown in a). c) Extracted line profile from b) along black line. d) FFT of the line profile from c). e) Microscope image (20x magnification) in bright field microscopy. Scale bar 100μm. f) Cryo-SEM of a WT IR1 colony from top view showing buckling. Scale bar 5 μm.

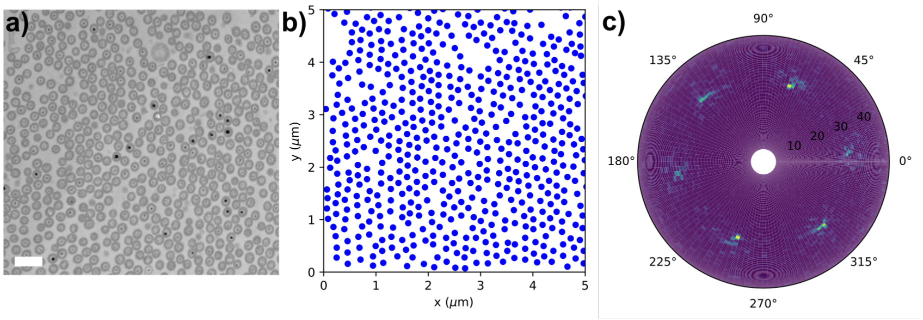

Figure S4: **Structure factor analysis:** a) TEM cross-section image of Flavobaterium strain IR1: scale bar 500 nm b) Extracted bacteria positions from a using ImageJ Software. c) K-space representation of b.

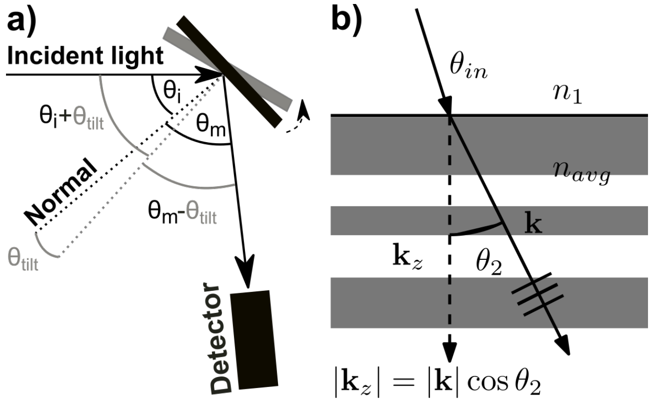

Figure S5: **Setup and sample sketches:** a) Sketch of tilting of the sample by θ_{tilt} with respect to the normal of the sample and its effect on the angular resolved measurement, b) Sketch of a multilayer structure showing all geometrical parameters involved in deriving eq. 2.

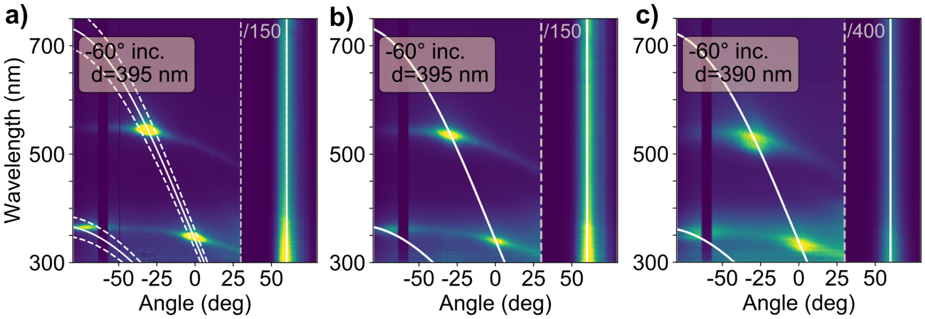

Figure S6: **Lattice constant reproducibility:** Measurements of three samples prepared as described in methods from a WT IR1 colony at day 2 showing the reproducibility and accuracy of the measurements and grating analysis (eq. 1, white lines). A variation in the lattice constant Δd±20 nm (white dashed lines in a) can not explain the broadening of both diffraction spots together.

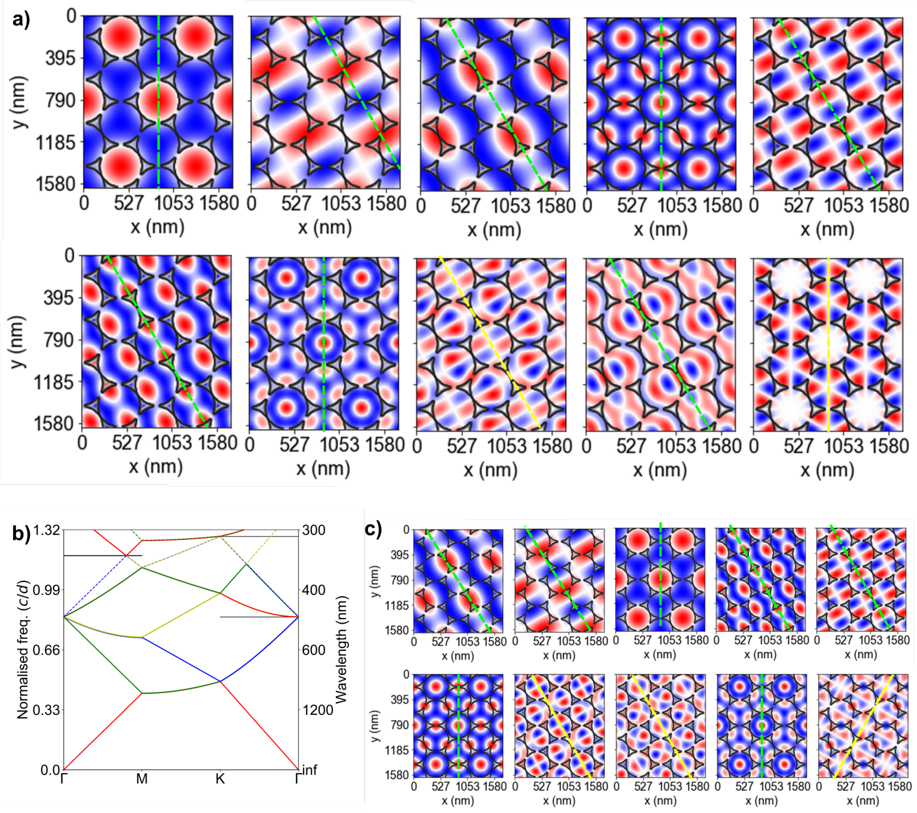

Figure S7: **Transverse Magnetic and Electric modes:** a) TM modes in an hexagonal crystal structure shown as black overlayed structure. b) Band diagram for TE modes. c) TE modes overlayed with scattering structure. Green lines indicate symmetries, yellow lines anti-symmetries.

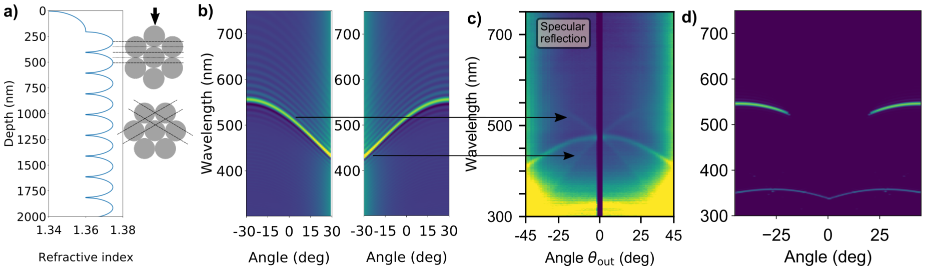

Figure S8: **Hexagonal crystal analysis:** a) Refractive index structure of a multilayer corresponding to a hexagonal crystal structure with incidence normal to the ΓM direction as visualized in the sketch on the right. The same multilayer appears twice when the crystal is tilted to incidence in the ΓK direction as pictured in the lower sketch. b) Result of the 1D transfer matrix simulation of the multilayer structure as pictured in a) tilted by 30 and -30 deg. c) Specular reflection measurement as in Fig. 3b but with rescaled intensities. d) First order FEM analysis indicating the appearance of the diffraction spot for varying incident angle.

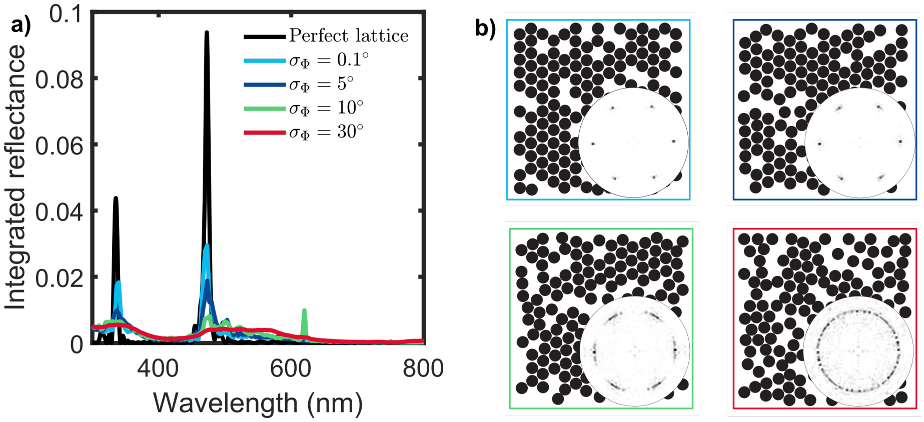

Figure S9: **Disorder analysis:** a) Same reflection spectra calculated by FDTD simulation as shown in Fig. 5d. b) Corresponding structures and angular space representations.
